# The effects of electrical stimulation of the peripheral vestibular system on neurochemical release in the rat striatum

**DOI:** 10.1371/journal.pone.0205869

**Published:** 2018-10-29

**Authors:** Lucy Stiles, Yiwen Zheng, Paul F. Smith

**Affiliations:** 1 Dept. of Pharmacology and Toxicology, School of Biomedical Sciences, University of Otago, Dunedin, New Zealand; 2 Brain Health Research Centre, University of Otago, Dunedin, New Zealand; 3 Brain Research New Zealand Centre of Research Excellence, University of Otago, Dunedin, New Zealand; 4 Brain Research New Zealand Centre of Research Excellence, University of Auckland, Auckland, New Zealand; 5 Eisdell Moore Centre for Hearing and Balance Research, University of Auckland, Auckland, New Zealand; University of North Carolina at Chapel Hill, UNITED STATES

## Abstract

For over a century, it has been speculated that the vestibular system transmits information about self-motion to the striatum. There have been inconsistent reports of such a connection, and interest in the subject has been increased by the experimental use of galvanic vestibular stimulation in the treatment of Parkinson’s Disease patients. Nonetheless, there are few data available on the effects of vestibular stimulation on neurochemical changes in the striatum. We used *in vivo* microdialysis to analyse changes in the extracellular levels of amino acids and monoamines in the rat striatum, following electrical vestibular stimulation. Stimulation caused a significant decrease in serine and threonine, compared to the no-stimulation controls (P ≤ 0.005 and P ≤ 0.01, respectively). The ratio of DOPAC:dopamine, decreased on the ipsilateral side following stimulation (P ≤ 0.005). There was a significant treatment x side x intensity interaction for taurine levels (P ≤ 0.002), due to a decrease on the contralateral side in stimulated animals, which varied as a function of current. These results show that peripheral vestibular stimulation causes some neurochemical changes in the striatum and support the view that activaton of the vestibular system exerts effects on the function of the striatum.

## Introduction

Numerous studies dating back to the early 20^th^ century, have suggested that the vestibular system might transmit sensory information concerning self-motion to the striatum, given the importance of the basal ganglia in the control of movement [1–3]. Potential pathways from the brainstem vestibular nucleus complex (VNC) or cerebellum, which receive primary afferent vestibular input, to the basal ganglia have been proposed, including via the motor cortex and the hippocampus [4]. More direct pathways from the VNC to the striatum, via the parafascicular nucleus (PFN) of the thalamus, have been supported by neurotracer and electrophysiological studies [5,6].

There have also been several electrophysiological studies conducted over the last few decades, in which electrical stimulation of the peripheral or central vestibular systems has been demonstrated to evoke field potentials in the striatum [7,8]. Very few single neuron recording studies have been conducted, and those published have yielded conflicting results, either showing selective responses to electrical vestibular stimulation [9]or not [10]. Nonetheless, Rancz et al. [11] reported that stimulation of the rat superior vestibular nerve could evoke field potentials and multi-unit activity in the rat striatum, a result that was confirmed using fMRI. In our recent study, we also found that a small population of striatal neurons responded to electrical stimulation of the rat peripheral vestibular system, in a phase-locked manner [12].These electrophysiological results are consistent with the results from PET and fMRI studies in humans, which have shown increases in activity in the striatum following either caloric or galvanic vestibular stimulation (GVS) [13–15].

Remarkably, there have been few studies of the effects of vestibular stimulation on neurotransmitter release in the striatum. Only one microdialysis study has been published, in which stochastic GVS for 30 min was shown to increase the release of GABA in the substantia nigra, but not in the striatum [16]; no significant changes in dopamine (DA), glutamate, aspartate, glycine, taurine, serine, alanine, 3,4-dihydroxyphenylacetic acid (DOPAC) or homovanillic acid (HVA) were observed. The only other published study, which used receptor autoradiography, showed that glutamic acid decarboxylase (GAD) levels increased in the striatum at 1 month following bilateral or unilateral vestibular deafferentation, suggesting an increase in GABA production [17].

In the entire basal ganglia, almost 99% of neurons have been identified as GABAergic [18], and in the striatum, all medium spiny neurons (MSNs) and all classes of interneuron besides one (the cholinergic interneurons), contain GABA. Dopaminergic innervation in the basal ganglia comes from two midbrain nuclei, the substantia nigra pars compacta (SNc) and the ventral tegmental area (VTA) [19,20].Projections from the SNc synapse primarily in the dorsal striatum [21], mostly on MSNs, where DA acts as a neuromodulator [22]. While DA and GABA are considered to be the major neurotransmitters in the striatum, a number of other neurochemicals play a role. Acetylcholine (ACh), from cholinergic interneurons, regulates MSN function by binding to muscarinic ACh receptors that are expressed extensively throughout the striatum, including on MSNs [23]. Serotonin [24,25], noradrenaline [26] and glycine [27], have all been found to play minor roles in striatal activity. Lesser studied amino acids have also been shown to be significant, including threonine, serine, and taurine [28–30].

Recently, interest in the possible connection between the vestibular system and the striatum has stimulated attempts to use stochastic GVS to treat the symptoms of Parkinson’s Disease (PD), where vestibular symptoms such as balance deficits are especially resistant to treatment [16,31,32]. Although the exact mechanism by which stochastic GVS exerts its effects on PD is unknown, Kim et al. [33]demonstrated that this form of electrical stimulation altered the beta and gamma bands of EEG in the brain, and suggested that it modulates the synchrony of multiple EEG oscillations. How this might specifically affect the striatum is unknown. Therefore, a better understanding of the potential effects of electrical stimulation of the vestibular system on neurochemical levels in the striatum, is urgently needed. The aim of this study was to use *in vivo* microdialysis to measure changes in the extracellular levels of a range of amino acids and monoamines in the striatum, in response to electrical stimulation of the rat vestibular system under anaesthesia. A total of 14 neurochemicals were initially analysed: 8 amino acids and metabolites (glutamate, serine, glutamine, glycine, threonine, alanine, taurine and GABA) and 6 monoamines and their metabolites (noradrenaline, dopamine, DOPAC, serotonin, 5-hydroxyindoleacetic acid (5-HIAA) and HVA). Amongst the amino acids, glutamate, GABA and glycine were measured because they are important neurotransmitters, and glutamine because it is a precursor to glutamate [16,34,35]. Serine can be converted to D-serine, which is regarded as a gliotransmitter and can modulate the N-methyl-D-aspartate (NMDA) subtype of glutamate receptor [29]. Taurine was measured because it is a neuromodulator that can act on GABA_A_ and GABA_B_ receptors [28,30,36]. Lastly, increased levels of threonine have been reported in PD patients [37]; therefore, it was also measured. Amongst the monoamines, noradrenaline, dopamine and serotonin were measured because they are also important neurotransmitters, and 5-HIAA and HVA/DOPAC are metabolites of serotonin and dopamine, respectively [16,17,19,20,22,25,26]. Based on previous electrophysiological studies in particular, it was predicted that there would be changes in the release of some of these neurochemicals in response to electrical stimulation of the vestibular system. Many of the neurochemicals investigated in this study are of direct relevance to PD. DA is, of course, the major neurotransmitter that is depleted in PD, and, as a result of this disinhibition, ACh levels are increased. Glutamate levels have been reported to increase in the striatum in PD [34], and GABAergic function becomes severely dysregulated with both an increase in the release of GABA and an increase in GABA_A_ receptor currents [35]. Glutamine would be expected to change also since it is a metabolite of glutamate, as would DOPAC and HVA, since they are metabolites of DA. There is very little evidence available on the relationship between taurine and PD; however, taurine levels have been reported to be correlated with the loss DA markers [36] and there has been unsubstantiated speculation that taurine may help to prevent the development of PD. Alanine and threonine levels have been reported to be higher in patients with PD compared to controls [37]. Finally, total D-serine content in the striatum has been shown to be increased in the 6-OHDA animal model of PD [38].

## Materials and methods

### Animals

Twenty male Wistar rats weighing between 250 and 350g were used for the microdialysis experiments. The animals were randomly divided into 3 groups: animals in which the striatum was sampled ipsilaterally to the electrical vestibular stimulation (n = 7), animals which were sampled contralaterally to the stimulation (n = 7), and a no stimulation control group (n = 6). Prior to the experiment animals were maintained on a 12 h light-dark cycle with free access to food and water. All procedures were approved by the University of Otago Animal Ethics Committee. All experiments were performed in accordance with relevant guidelines and regulations.

### Stimulating electrode implantation

The animals were anaesthetised with urethane (1.5 g/kg, i.p) and were considered ready for surgery when the pedal-withdrawal reflex was absent. During surgery, the animals’ body temperatures were monitored using a rectal probe (Harvard Apparatus) and maintained at 37°C. Xylocaine (with 1:10,000 adrenaline; 0.05 ml, s.c) was injected around the wound margins before any incisions were made.

**Fig 1:**
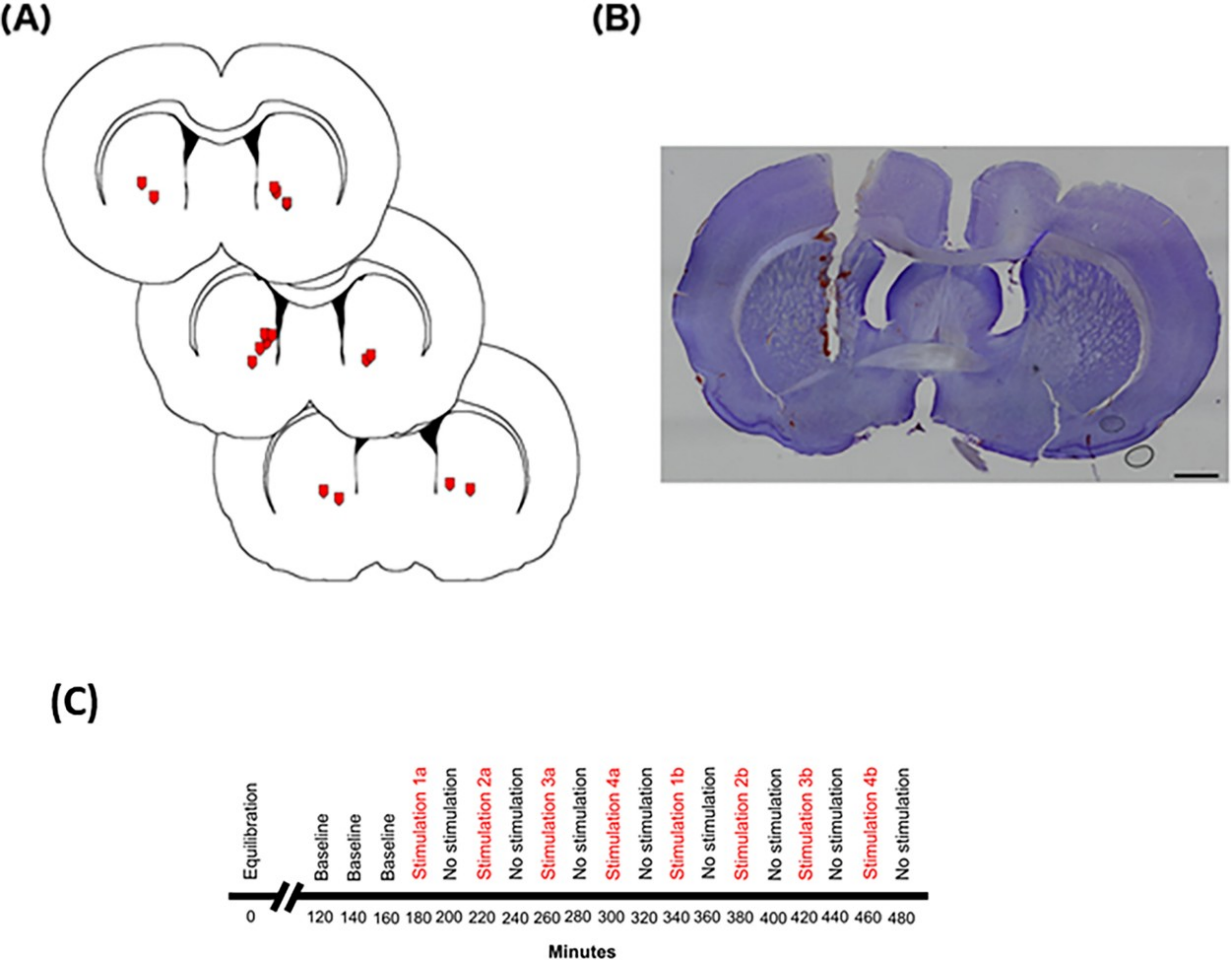
**(A)** Location of microdialysis probe tips the in striatum. Microdialysis probe locations (red symbols signify tip location) were seen between 0.50 mm and 1.20 mm from bregma based on Paxinos and Watson^**47**^. Probe membranes measure 2 mm upward from the tip of the probe. **(B)** Cresyl violet-stained tissue showing a microdialysis probe track through the cortex and into the striatum. Scale bar represents 1000 μm. **(C)** Timeline of microdialysis sampling. A 2 μm microdialysis probe was inserted into the striatum of urethane-anaesthetised rats. The timeline shows min following probe insertion. Artificial cerebrospinal fluid was perfused through the probe at a rate of 1.5 μl/min for the entirety of the sampling time. After 2 h of equilibration samples were collected every 20 min under different conditions.

**Fig 2:**
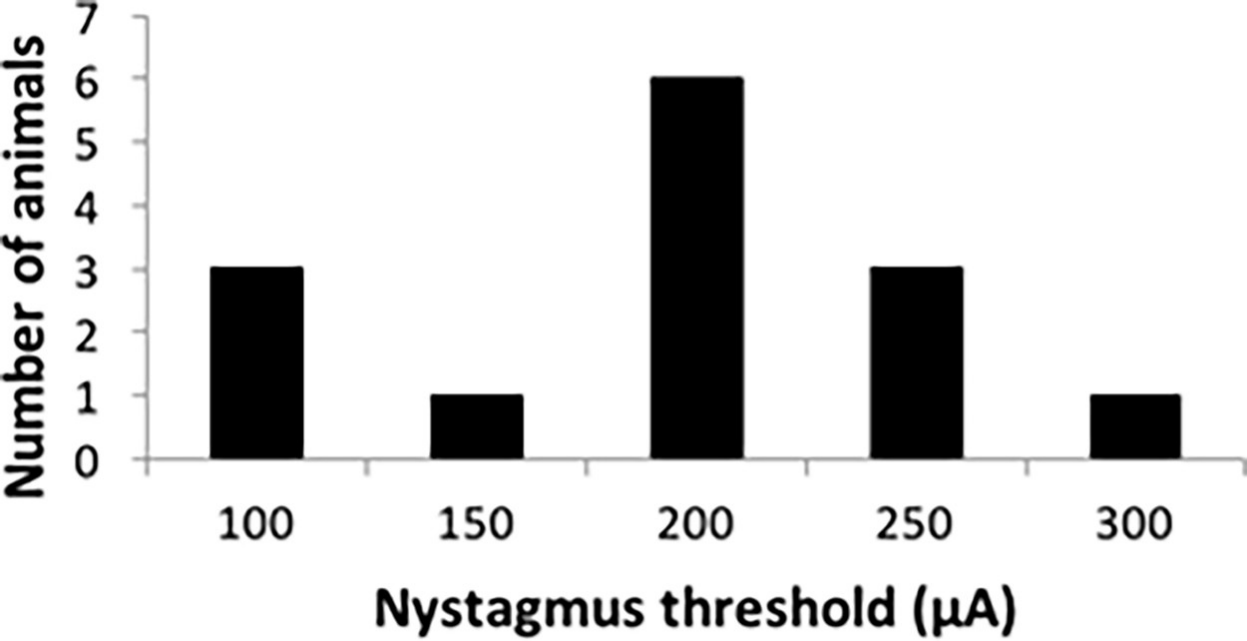
Thresholds of nystagmus for the animals used in the microdialysis study. Data are shown as number of animals (n = 14) with thresholds as specific amplitudes (μA).

The vestibular stimulating electrode was bipolar and was implanted into the round window, under an otolaryngological microscope (OPMI Pico, Zeiss, Hamburg, Germany). The tympanic bulla was exposed using a retro-auricular surgical approach and a dental drill used to open the bulla to expose the round window. A stainless-steel bipolar electrode (MS303/1-B/SPC, Plastics One Inc.), insulated except at the tip, was placed into the round window as the stimulating electrode. It was secured in place using dental cement once the location of the electrode placement was confirmed via visualisation of vestibular nystagmus in response to stimulation, which was considered evidence that the peripheral vestibular system was being adequately activated [12,39] (Fig 1C). The surgical site was then sutured closed. Eye movements were recorded using a Dino-Lite microscopic video camera focused on the rat’s eye [12,39], images from which were displayed on a PC monitor. The threshold for nystagmus was defined as the lowest current at which eye movement was visible (Fig 2). Analysis of the video files of the animals’ eye movements was performed using eye tracking software (AET Tracker, STARNAV, France). Electrical stimulation was controlled using the Spike-2 software package and the stimulation current was produced using an analogue stimulation isolator (Model 2200: A-M systems). No stimulation animals received the same surgery but without an electrode implanted. The stimulation was applied for 20 min in each case, based on the results we obtained in our previous study using single neuron recording and c-Fos expression in the striatum [12]. Microdialysis probe implantation occurred within 1 h of implanting the stimulating electrodes, in order to ensure that the stimulating electrode placement did not change.

### Microdialysis probe preparation and implantation

New microdialysis probes (CMA 12 Elite probe, 2 mm, CMA microdialysis) were primed for use by running filtered (22 μm, durapore PVDF syringe filter, Millipore) artificial cerebrospinal fluid (aCSF; 125 mM NaCl, 3 mM KCl, 1.2 mM MgSO_4_∙7H_2_O, 1.2 mM CaCl_2_∙2H_2_O, 0.5 mM NaH_2_PO_4_∙H_2_O, 5 mM Na_2_HPO_4_, pH 7.4) through the probe at 12 μl/min for 5 min, in order to remove any air bubbles from the probe. After priming, as well as after each microdialysis experiment, a recovery assay was performed to determine the recovery rate of the probe for each chemical by immersing the probe in a medium solution containing known concentrations of each of the neurochemicals to be analysed and comparing the concentration of the neurochemicals in the microdialysate with that in the medium solution.

The animal was transferred to a stereotaxic frame with ear bars for the microdialysis probe implantation. Xylocaine (with 1:10,000 adrenaline; 0.05 ml, s.c) was injected along the midline of the head before an incision was made. Using a dental drill, a small hole was made in the skull to unilaterally expose the area of the brain containing the striatum, either the ipsilateral or contralateral hemisphere to the vestibular stimulating electrode (0.5 mm from bregma and 2–3 mm lateral to the midline) [12,40]; the dura was pierced. A guide cannula (CMA 12 Guide Cannula, CMA microdialysis) was inserted into the brain 6 mm from the top of the skull at a rate of 4 μm/sec. The cannula was then retracted to a depth of 4 mm; this was to allow for the 2 mm membrane of the probe to extend from the end of the cannula. Previous studies have shown that the implantation of a microdialysis probe can result in the release of neurotransmitters that is not dependent on neuronal activation [41,42]. However, Borland *et al* [43] demonstrated that by implanting at a rate of approximately 4 μm/sec, DA levels in the dialysate were stable almost immediately after implantation. Furthermore, 2 h after implantation, Borland *et al*. [43] found that the release was largely tetrodotoxin-sensitive, implying that any DA release was due to synaptic release and not tissue damage. The probe was connected to a 1 ml Hamilton syringe containing filtered aCSF in a syringe pump and then inserted into the guide cannula. The syringe pump was set to a flow rate of 1.5 μl/min and the probe was equilibrated in place in the brain for 2 h before samples were collected.

### Microdialysis sampling protocol

Following equilibration, 30 μl of dialysate was collected on ice for each sample collection (20 min per sample), which was then stored at -80°C for further analysis. Three initial baseline samples were collected and then a non-stimulation sample was collected between each stimulation sample. Stimulation was delivered at 4 current intensities (0.5x, 1x, 2x, and 3x the threshold of nystagmus). Time was allowed for the clearance of the outflow tube in between samples to ensure that the sample collected contained only fluid sampled during either the stimulation or non-stimulation periods and there was no cross-over between samples. To avoid any kind of order effect, the 4 intensities of stimulation were delivered in a random order and that order was repeated twice. This resulted in a total of 8 stimulation samples collected and 11 non-stimulation samples collected (Fig 1C). The objective of delivering the same 4 stimulus currents to each animal was to make it possible to compare the effects of the current amplitudes within the same animals, taking advantage of the smaller variability within a particular animal, compared to between animals. The use of a random order was intended to control for the possibility of order effects caused by the same order of currents in each case. It is conceivable that the use of multiple currents obscured the effects of the proceeding current; however, the only alternative to this would have been to use separate groups of animals for every current, requiring 4 times the number of animals. The fact that the responses to the different currents were comparable does not prove, but suggests to us, that there might have been minimal effect of the previous current on the following current in each set of 4 currents. An indication that the effects of the vestibular stimulation did not saturate, was that nystagmus could still be evoked at the end of the complete stimulation protocol [12]. The same kinds of samples were collected for the no stimulation animals; however, no stimulation was given. Although 20 min sampling periods would not be sufficient to detect instantaneous changes, we reasoned that they should detect cumulative changes, and previous studies using 20 min sampling periods support this [44]. It is also possible that the process of implanting the microdialysis probes into the striatum resulted in non-specific effects on neurotransmitter release. However, the purpose of the non-stimulation controls, which also had the microdialysis probes implanted into the striatum, was to control for this. It is possible that some of the variability in neurochemical release prior to stimulation was due to such non-specific effects. However, such effects were considered to be controlled for in the design of the experiment by both normalizing the post-stimulation neurochemical levels to baseline and by comparing the stimulation animals with the no stimulation animals.

### Histological analysis for probe placement

At the completion of the experiment the animal was sacrificed via cervical dislocation and the whole brain was removed and placed in 10% formalin solution. Two days before sectioning, the brain was transferred to a 30% sucrose in phosphate buffer (PB) solution. The brains were refrigerated at 4°C prior to sectioning. The tissue was cut into 40 **μ**m sections using a freezing microtome. Sections were collected in 0.01 M phosphate buffered saline (PBS) in 24 well plates. Cresyl violet staining was used to identify brain structures and aid in the visualization of the location of probe tracks in the striatum (Fig 1). Sections were incubated with 0.0015% cresyl violet diluted in acetic acid for 30 min and were transferred to slides and dried. Sections were then dehydrated in increasing concentrations of ethanol: (50% - 100%), cleared in xylene and coverslipped.

### High-performance liquid chromatography (HPLC) and electrochemical detection (ECD)

Microdialysis samples were analysed using HPLC (Hewlett Packard 1100 series) with ECD (ECD-3000RS Electrochemical Detector, Thermo Scientific). Amino acids in the standards and microdialysis samples were derivatised pre-column with an *o*-phthaldialdehyde (OPA)/β-mecaptoethanol (BME) reagent. Briefly, 5 **μ**l of each standard solution or microdialysis sample was mixed with 10 **μ**l of internal standard, homoserine, and 40 **μ**l OPA/BME reagent by the autosampler and the derivatisation reaction allowed to occur for 2 min at 8°C before sample injection. Amino acids were separated on a Luna C18(2) column (3 μm, 100 Å, 100 x 4.6 mm, Phenomenex Inc.) using a mobile phase containing 0.1 M Na_2_HPO_4_, 23% methanol and 7% acetonitrile (pH 6.75) with a flow rate of 0.85 mL/min. The amino acids’ detection was carried out by the ECD using the settings of E1: 650 mV, 100 nA; E2: 200 mV, 100 **μ**A; and E3: 600 mV, 500 nA. As baseline levels of GABA are very low, attempts were made to increase the sensitivity of GABA detection by reducing the retention time for GABA through optimising the mobile phase. The final mobile phase composition used for the detection of GABA was 0.1 M Na_2_HPO_4,_ 12% methanol and 18% ACN (pH 6.25).

Monoamines were separated on a Luna C18(2) column (3 μm, 100 Å, 150 x 4.6 mm, Phenomenex Inc.) using a mobile phase containing 31 mM citric acid monohydrate, 50 mM Na_2_HPO_4_, 0.13 mM ethylenediaminetetraacetic acid (EDTA), 0.576 mM 1-octane sulphonic acid, 0.001% triethyamine and 15% methanol at pH 3.0 with a flow rate of 1.0 mL/min. The detection conditions for the ECD were set at E1: 400 mV, 1 **μ**A; E2: -150 mV, 100 nA; and E3: 350 mV, 100 nA. N^ω^-methylserotonin was used as the internal standard. Briefly, each standard solution or microdialysis sample was mixed with an equal volume of internal standard at 4°C and 15 **μ**l of the mixture was injected onto the column by the autosampler.

### Data analysis and statistical analyses

Neurochemicals were identified based on their retention times, and the concentrations of the neurochemicals in the dialysates were calculated based on the analyte:internal standard peak area ratios, using the calibration curves established with the standards used for each individual neurochemical that were included in each assay using the Chromeleon software (Thermo Scientific). The recovery rate of the microdialysis probe was obtained for each neurochemical by calculating the ratio between the concentration measured in the recovery dialysate and the concentration in the medium solution, and expressed as a percentage. To calculate the amount of each individual neurochemical in the brain during each sampling period, the concentrations obtained from the HPLC analysis were divided by the recovery rate of the probe prior to the microdialysis, as described in the following equation:

Concentrationinthebrain=(dialysateconcentration/recoveryrate)x100

The data were analysed statistically in 2 different ways. First, levels of the neurochemicals during the non-stimulation periods (see Fig 1C) were compared between the stimulation and no stimulation groups in order to determine whether the level of neurochemical release changed over time. Second, the levels of the neurochemicals during the stimulation periods were analysed as a % of the initial baseline for the no stimulation and stimulation animals in order to determine whether neurochemical release changed as a result of the stimulation. Since each sequence of stimulations was run twice, the results of both stimulation sequences were expressed as a % of the initial baseline. Normalising the data to the baseline allowed for a comparison of responses to the stimulation that controlled for variations in the neurochemical levels between individual animals. The initial baseline was calculated by averaging the 3 baseline samples. Percentages were then calculated as a percentage of this average baseline.

Further analysis was performed to compare the ratios of the neurotransmitters to their metabolites in order to find out whether the metabolism of any of the neurotransmitters had changed in response to the stimulation. This included the ratios of: glutamine to glutamate, DOPAC to DA, and HVA to DA. These data were also analysed the same way as the individual neurochemicals.

Analysis of the non-stimulation raw data, as well as the non-stimulation metabolite ratios, was performed using a 2 factor Linear Mixed Model (LMM) analysis in SPSS 24, in order to control for the extensive correlation in repeated measures data [45]. The data were tested for normality and transformed when necessary. The Akaike’s Information Criterion (AIC) was used to select the optimal covariance matrix structure. Bonferroni post-hoc tests were used in the case of significant factors in the LMM. The stimulation data were analysed using generalized estimating equations (GEE) within the generalized linear mixed model (GLMM) analysis option in SPSS 24, with the stimulation and side as between-group factors and intensity as a repeated measure [45]. The GEE-GLMM was used in preference to LMM analysis because the data were found to be extremely non-normally distributed and this could not be corrected with data transformation. The Corrected Quasi-Likelihood Criterion (QICC) was used to determine the goodness of fit of the covariance matrix structures^45^. Where there were significant effects, Bonferroni post-hoc tests were carried out [45].

## Results

The experiments were designed so that baseline measurements (i.e., non-stimulation periods) were made first, and then 4 different current amplitudes, defined in relation to their ability to activate the vestibular system (Fig 1C; Fig 2), were used to determine whether vestibular stimulation altered neurochemical release (i.e., stimulation periods). In each case the order of the different current amplitudes was pseudo-randomised in order to avoid order effects, and the change in neurochemical level was expressed as a % of the initial baseline, in order to control for fluctuating baselines (Fig 1C). Microdialysis sampling was conducted on the same side as the vestibular stimulation (ipsilateral), or the opposite side (contralateral), and compared to ipsilateral and contralateral no stimulation control groups. The metabolism of the neurochemicals was also investigated by determining the ratios of the neurochemicals to their metabolites, e.g. DOPAC:DA.

### Location of microdialysis probes

The microdialysis probes were located mostly in the medial striatum (Fig 1A and 1B). The tip of one probe was found to be located in the lateral ventricle and therefore this animal (ipsilateral stimulation group) was excluded from further analysis.

### HPLC-ECD output

**Fig 3:**
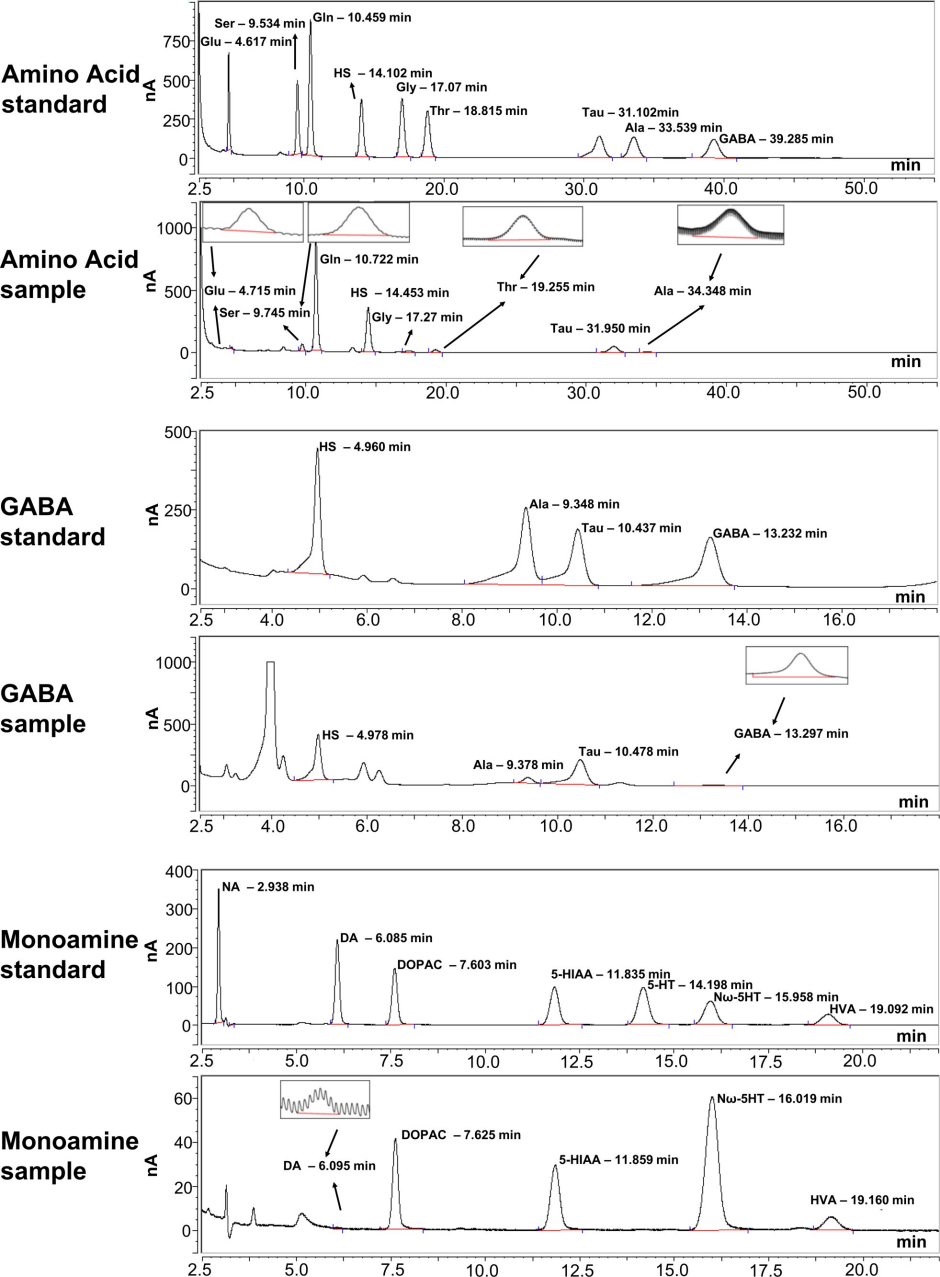
Example traces of HPLC-ECD output. Traces show peaks for standards and samples for amino acid (top), GABA (middle), and monoamine (bottom) analysis. Retention times for peaks were consistent between samples and experiments. Insets show analysable peaks of low concentration neurochemicals. Glu–glutamate; Ser–serine; Gln–glutamine; HS–homoserine; Gly–glycine; Thr–threonine; Tau–taurine; Ala–alanine; NA–noradrenaline; DA–dopamine; DOPAC– 3,4-dihydroxyphenylacetic acid; 5-HT–serotonin, 5-HIAA– 5-hydroxyindoleacetic acid; Nω-5HT–Nω-methylserotonin; HVA–homovanillic acid.

HPLC-ECD analysis produced clear detection peaks and consistent retention times for each of the neurochemicals (Fig 3). Recovery analysis showed that the recovery rates of serotonin and 5-HIAA in the stock solution (200 pg/μl) were negligible. Recovery at a higher concentration (20 ng/μl) did reveal the neurochemicals at measurable concentrations, suggesting that while the probes were able to collect those neurochemicals, in the diasylate samples they were too low to be detected. Levels of noradrenaline in the diasylate samples were also too low to be detected by the HPLC-ECD method used. Glycine levels in the diasylate were contaminated by another peak of an unknown chemical. Due to the issues with these neurochemicals, they were excluded from further analysis.

### Extracellular levels of neurochemicals in the striatum during the non-stimulation periods

**Fig 4:**
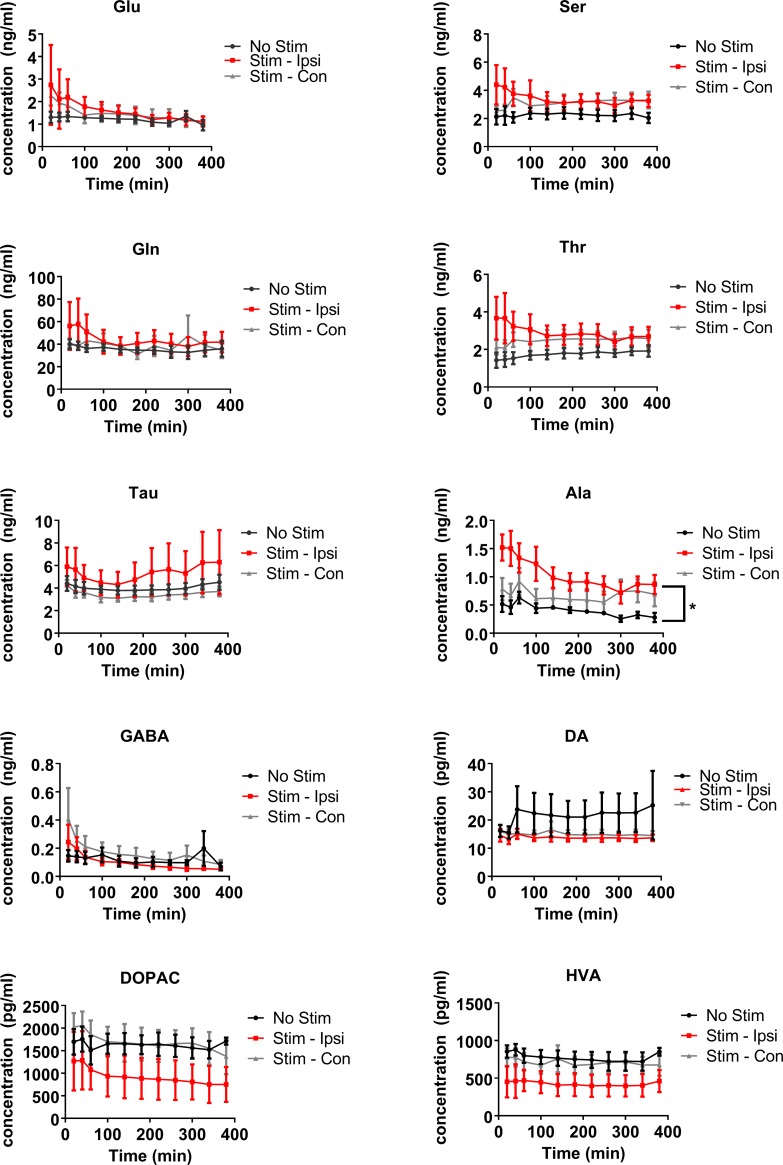
Changes in the levels of neurochemicals of non-stimulation samples in the striatum of rats that were allocated to either the electrical vestibular stimulation or no stimulation treatment groups. Glu–glutamate; Ser–serine; Gln–glutamine; Thr–threonine; Tau–taurine; Ala–alanine; DA–dopamine; DOPAC– 3,4-dihydroxyphenylacetic acid; HVA–homovanillic acid. Stim—Ipsi–samples collected on the side ipsilateral to stimulation; Stim—Con–samples collected on the side contralateral to stimulation. Data points are represented as mean ± SEM. Only significant main treatment effects, i.e. due to stimulation alone, are shown for clarity (i.e. no interactions). * = P ≤ 0.05.

Alanine (F(2) = 4.58, P ≤ 0.05) was the only neurochemical to show a significant group difference during the non-stimulation periods, with alanine release on the side that would be ipsilateral to stimulation, higher than that seen on the contralateral side or in the no-stimulation animals (pairwise comparison: P ≤ 0.05) (Fig 4). With respect to time, there were changes in the neurochemical levels of non-stimulation samples for glutamate (F(10) = 3.41, P ≤ 0.005), taurine (F(10) = 4.34, P ≤ 0.0001), alanine (F(10) = 3.79, P ≤ 0.005) and GABA (F(10) = 2.83, P ≤ 0.01; Fig 4). It appeared that the changes over time were due to higher levels of the neurochemicals at the beginning of the sampling period, possibly due to variations in the exact probe placement. A time x treatment group interaction was seen in relation to GABA release (F(2,10) = 2.08, P ≤ 0.02), showing that GABA levels in both the ipsilateral and contralateral stimulation groups were higher at the start than those in the no-stimulation animals. There were no other significant interactions seen in the levels of the non-stimulation samples.

### Extracellular levels of neurochemicals in the striatum during the stimulation periods

**Fig 5:**
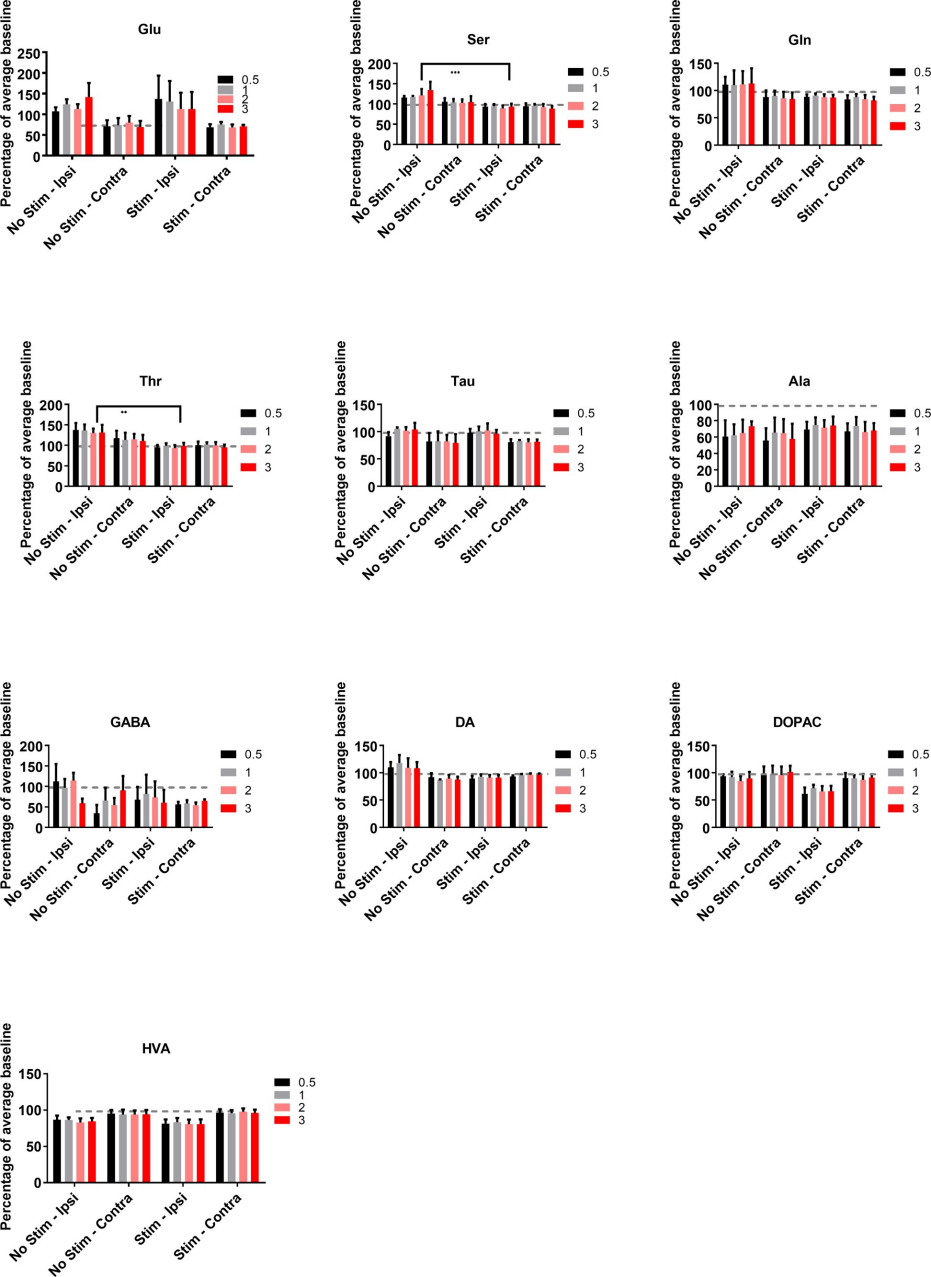
Changes in neurochemical levels in the striatum of rats that received either electrical vestibular stimulation or no stimulation treatment, normalised to the average of the initial baseline. Glu–glutamate; Ser–serine; Gln–glutamine; Thr–threonine; Tau–taurine; Ala–alanine; DA–dopamine; DOPAC– 3,4-dihydroxyphenylacetic acid; HVA–homovanillic acid. Stim—Ipsi–samples collected on the side ipsilateral to stimulation; Stim - Contra–samples collected on the side contralateral to stimulation. Data points are represented as mean ± SEM. Only significant main treatment effects, i.e. due to stimulation alone, are shown for clarity (i.e. no interactions). ** = P ≤ 0.01; *** = P ≤ 0.005.

Changes in the stimulus-dependent release of the neurochemicals were analysed as a % of the averaged initial baseline for the no-stimulation and stimulation animals. Electrical stimulation was found to cause statistically significant changes in the neurochemical levels of serine (Wald χ2 (1) = 9.14, P ≤ 0.005) and threonine (Wald χ2 (1) = 6.73, P ≤ 0.01). Both neurochemicals exhibited decreases in the stimulated samples versus the no-stimulation samples (Fig 5). The levels of DA were found to be significantly higher on the ipsilateral side of no-stimulation animals compared to the other groups (treatment x side interaction: Wald χ2 (1,1) = 4.94, P ≤ 0.05); however, the post-hoc tests showed that there were no significant pairwise differences. There was also a significant side x intensity interaction for DA (Wald χ2 (1,3) = 8.23, P ≤ 0.05) (Fig 5).

The intensity of the stimulation was found to have a significant effect on the levels of glutamate (Wald χ2 (3) = 10.32, P ≤ 0.02), glutamine (Wald χ2 (3) = 9.14, P ≤ 0.05), taurine (Wald χ2 (3) = 13.98, P ≤ 0.05), and alanine (Wald χ2 (3) = 11.59, P ≤ 0.01). Significant side differences were found in the levels of glutamate (Wald χ2 (1) = 4.93, P≤ 0.05), taurine (Wald χ2 (1) = 5.04, P≤ 0.05), and HVA (Wald χ2 (1) = 6.92, P≤ 0.01). However, only taurine levels were found to have significant side x intensity (Wald χ2 (1,3) = 13.13, P ≤ 0.005) and treatment x side x intensity interactions (Wald χ2 (1,1,3) = 14.47, P ≤ 0.002). Post-hoc tests revealed significant pairwise differences for the ipsilateral no-stimulation group compared to the contralateral stimulated groups at intensities of 0.5, 1, 2 and 3x the nystagmus threshold, with taurine levels decreasing following stimulation (P ≤ 0.001 for 0.5, 1x, 2x and P ≤ 0.0001 for 3x). A significant interaction was also found for side x intensity for alanine (Wald χ2 (1,3) = 9.61, P ≤ 0.05; Fig 5); however, the only significant pairwise post-hoc comparison was between the contralateral side at the 1x intensity and the contralateral side at the 3x intensity (P ≤ 0.0001).

### Metabolism of neurochemicals in the striatum during the non-stimulation periods

**Fig 6:**
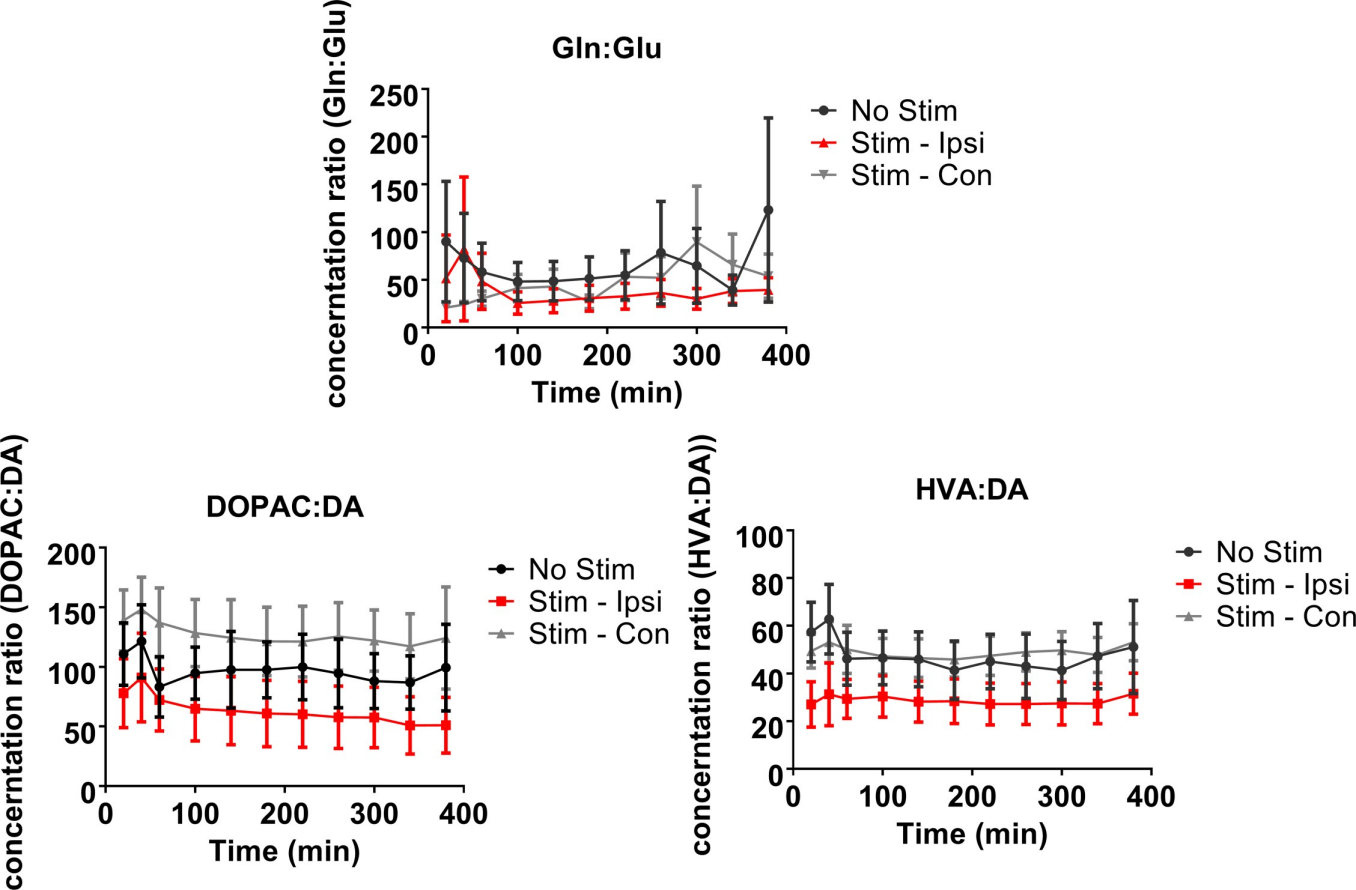
Changes in non-stimulation levels of neurochemical metabolite ratios in the rat striatum following electrical vestibular stimulation. Glu–glutamate; Gln–glutamine; DA–dopamine; DOPAC– 3,4-dihydroxyphenylacetic acid; HVA–homovanillic acid. Stim—Ipsi–samples collected on the side ipsilateral to stimulation; Stim—Con–samples collected on the side contralateral to stimulation. Data points are represented as mean ± SEM.

The ratios of the neurotransmitters to their metabolites were analysed in order to see how neurotransmitter turnover might have changed in response to electrical vestibular stimulation. During the non-stimulation period, the ratios of glutamine:glutamate (F(10) = 3.10, P ≤ 0.005) and HVA:DA (F(10) = 2.04, P ≤ 0.05; Fig 6) changed significantly with time. The ratio between glutamine:glutamate also showed a significant interaction between stimulation and time (F(2,10) = 1.94, P ≤ 0.05; Fig 6). The ratio between HVA:DA showed no significant changes in the pairwise comparisons.

### Metabolism of neurochemicals in the striatum during the stimulation periods

**Fig 7:**
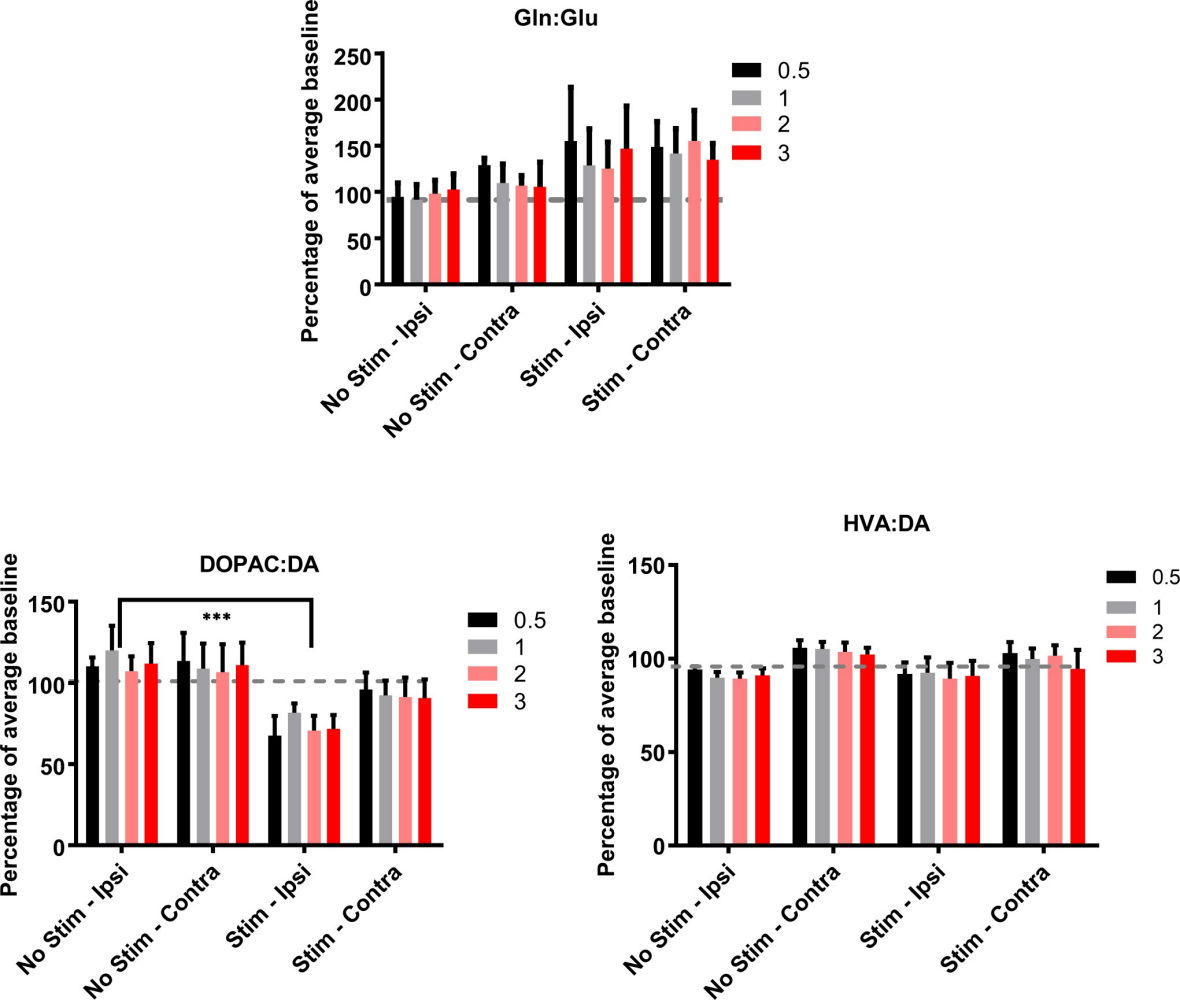
Changes metabolite ratios in the rat striatum in response to electrical vestibular stimulation normalised to the average of the initial baseline. Glu–glutamate; Gln–glutamine; DA–dopamine; DOPAC– 3,4-dihydroxyphenylacetic acid; HVA–homovanillic acid. No stimulation—Ipsi–samples collected on the side ipsilateral to no stimulation; No stimulation - Contra–samples collected on the side contralateral to no stimulation. Stim—Ipsi–samples collected on the side ipsilateral to stimulation; Stim—Contra–samples collected on the side contralateral to stimulation. Data points are represented as mean ± SEM. Only significant main treatment effects, i.e. due to stimulation alone, are shown for clarity (i.e. no interactions). *** = P ≤ 0.005.

Percentage changes were calculated for the stimulus-dependent release of the neurochemicals normalised as the % of the initial baseline for the no-stimulation and stimulation animals and then the metabolic ratios were calculated. Electrical stimulation of the vestibular system resulted in significant changes in the DOPAC:DA ratio (Wald χ2 (1) = 8.644, P ≤ 0.005; Fig 7). This appeared to be due to a decrease in the ratio on the side ipsilateral to the stimulation versus the ipsilateral no-stimulation group. The intensity of the stimulation was found to have a significant effect on the ratio of glutamine:glutamate (Wald χ2 (3) = 10.46, P ≤ 0.02), due to an increase in the ratio at the 0.5x intensity compared to the other stimulation levels. A significant side difference was found for the ratio of HVA:DA, with the ratio on the contralateral side higher than that on the ipsilateral side for both the no-stimulation and stimulation groups (Wald χ2 (1) = 4.73, P≤ 0.05).

## Discussion

To the best of our knowledge, this is only the second study to use *in vivo* microdialysis to measure the effects of electrical stimulation of the peripheral vestibular system in rat on neurotransmitter release in the striatum. The first one was by Samoudi et al. [16], who investigated the effects of stochastic GVS for 30 min on GABA and DA release in the striatum and SN. However, stochastic GVS, which superimposes a Gaussian noise signal over the stimulus, is quite a different method of electrical stimulation, intended to mimic the effects of similar stimulation in PD patients, rather than as a means of mapping the effects of activation of the vestibular system on the basal ganglia.

We found limited changes in the neurochemicals we investigated in the striatum, in response to vestibular stimulation which evoked vestibular nystagmus. Changes in the stimulus-dependent release of the neurochemicals were analysed as a % of the averaged initial baseline for each of the no-stimulation and stimulation animals. Only serine and threonine showed significant decreases in the stimulated animals, compared to the no-stimulation controls, that were independent of side and/or current intensity. DA was found to show a small but significant decrease in release, on the side ipsilateral to stimulation compared to the ipsilateral no-stimulation control group. However, the levels of DA in the contralateral no-stimulation and contralateral stimulated groups, were also lower than in the ipsilateral no-stimulation control group and there were no significant post-hoc pairwise comparisons. Therefore, there was no clear evidence that the decrease in DA in the ipsilateral stimulated animals was specific to that group. However, further analysis of the ratio of DOPAC to DA revealed a significant decrease in the ipsilateral striatum following stimulation. The DOPAC to DA ratio has been used as a measurement of DA metabolism [46,47]. The decreased DOPAC/DA ratio in the present study may suggest a reduced DA metabolism following vestibular stimulation. It has been shown that stimulation of the subthalamic nucleus (STN) increases DA metabolism, as indicated by an increase in both the HVA/DA and DOPAC/DA ratios [48]. However, the same stimulation caused a decrease in DA metabolism in 6-OHDA-lesioned rats [48]. It was speculated that this decrease in DA metabolism following stimulation might have served as a mechanism to maintain DA levels in 6-OHDA lesioned rats. In addition, activation of DA D_1_ receptors increases the DOPAC to DA ratio, while activation of the D_2_ receptor decreases it [46]. Therefore, a reduced DA metabolism could be a result of reduced activation of the DA system or a response to maintain DA activity. Further studies to correlate DA metabolism with neuronal activity may provide insight into how the DA system is affected by vestibular stimulation. It is of interest to note that Samoudi et al. [16], using stochastic GVS, found no significant effects on DOPAC in the striatum. However, as mentioned earlier, stochastic GVS is a very different stimulus to the one used here.

Taurine levels also exhibited a significant decrease on the contralateral side in stimulated animals. Taurine is an aminosulfonic acid, which is considered to be a neuromodulator and is known to mediate some of its effects via GABA_A_ and GABA_B_ receptors [49]. As with serine and DOPAC, Samoudi et al. [16] found no significant effects on taurine in the striatum using stochastic GVS.

It was interesting that the levels of serine and threonine were found to be lower in the stimulated animals than the no-stimulation animals. Serine is a neuroactive chemical when found in the dextrorotation (D-serine) form. While L-serine is the predominant form in the body, it can be converted to D-serine by serine racemase in astrocytes [50,51]. D-serine is classed as a gliotransmitter, as it is released from astrocytes in a calcium-dependent manner [52]. It binds to the glycine binding site on NMDA receptors and causes receptor activation [52] While the role of serine in the striatum is unknown, it has been demonstrated that serine is necessary for the development of long-term potentiation [53,54]. From this it can be hypothesised that serine may play a role in movement initiation and motor learning [53,54,55]. While the isomers were not analysed here, it can be assumed that at least some of the serine measured was D-serine and may therefore have affected NMDA receptor activity. Threonine has been assumed to be a non-neuroactive amino acid; however, increased levels of threonine have been reported in PD patients [37].

Most of the microdialysis probes were positioned quite ventrally in the striatum (see Fig 1B), and therefore most of the DA in this area would probably arise from the VTA. The nucleus accumbens and the olfactory tubercle comprise the ventral striatum, which has specific roles in cognitive functions related to motivation, aversion and reward [56]. Consequently, the effects of vestibular stimulation on neurochemical release in this particular area of the striatum could be quite different from other areas such as the dorsal striatum.

It is critical in microdialysis studies to normalise the effects of any stimulation on neurochemical release, to a pre-stimulation baseline, and in this case all of the data collected in response to stimulation (or no-stimulation) were expressed as a % of these baselines. However, in addition, we also analysed the stability of the neurochemical levels before stimulation, in order to determine whether they changed spontaneously as a result of time. Indeed, we did find significant changes in the non-stimulation samples for alanine, GABA, glutamate, and taurine, which appeared to be due to changes in the baseline levels in the animals allocated to the stimulation group, but before the stimulation. For GABA, there was a significant interaction between group allocation and time, which might have been due to the electrical stimulation that the animals received during the placement of the stimulating electrode, prior to microdialysis. It is important to keep in mind, when interpreting the effects of the vestibular stimulation, that neurochemical levels may change over time anyway, even without intervention, simply as a result of changes in the animal’s brain activity over time. Such stimulation-independent changes can occur for the neurochemical-metabolite ratios as well, and we also found that time was a significant factor for the glutamine-glutamate ratio. It is for this reason that we expressed all of the stimulation data as a % of the baseline data, in order to control for differences in baseline levels. While some baselines did vary over time, the differences that we detected as a result of vestibular stimulation should have been superimposed on those differences.

In general, it would be expected that the intensity of the stimulation current would have a significant effect on neurochemical release, and this was the case for glutamate, glutamine, taurine, and alanine, with alanine also showing a significant intensity effect in interaction with side and taurine levels, and a significant treatment x side x intensity interaction. It was notable, however, that for most of the neurochemicals for which changes were detected as a result of the stimulation, these changes did not vary much as a function of the different currents used and in most cases the differences could be seen even at 0.5x the threshold for nystagmus. This suggests to us that these effects could be produced even at low current amplitudes that were likely to be very selective for vestibular activation, given the use of bipolar electrodes in the round window.

It is possible that the implantation of the microdialysis probes and the stimulation prior to microdialysis sampling may have had an effect on the non-stimulation samples taken. This could be a major explanation of the inter-animal variability observed prior to stimulation. Despite accurate stereotaxic coordinates, it was inevitable that the microdialysis probes would be located in slightly different positions in the striatum. It was expected from the results of our electrophysiological studies [12] that following the end of the stimulation, the stimulated neurons would cease firing and the extracellular neurochemicals would be then rapidly taken up by neurons and glia. Therefore, the neurochemical levels would return to baseline rapidly following stimulation. In order to control for any type of order effect that the different stimulation intensities might have, they were applied in a pseudo-random order. However, while this meant that the order of the stimulations was controlled for, it limited the ability to analyse the effects of the different stimulation intensities on the subsequent non-stimulation samples. It is also possible that the implantation of the microdialysis probes caused an immunological reaction that confounded the results. For example, Woodroofe et al. [57] demonstrated that microdialysis probe implantation in the brain resulted in the increased production of interleukins -1 and -6 by macrophages during a 24 to 48 h period following probe implantation. However, the timescale of our experiments after implantation was approximately 10 hs, long before this kind of immune response would have occurred. Nonetheless, other rapidly occurring immune responses cannot be excluded, and neither can plasticity mechanisms that might have been stimulated by the trauma caused by the probe implantation. However, since similar probe implantation occurred in both the stimulation and no stimulation groups, it is difficult to ascribe any effects of the stimulation to the probe placement in the stimulation group alone.

ACh is a significant neurotransmitter in the striatum, which was not analysed in the present study due to the difficulty in collecting enough sample volume for all of the analyses. ACh analysis via HPLC requires a unique protocol in addition to those performed above [58]. Therefore, to acquire enough sample volume to analyse ACh, in addition to the other neurochemicals, would have either required a stimulation period long enough to potentially cause sensitisation, or a higher microdialysis flow rate, which would have significantly decreased the neurochemical recovery rate. As there are known changes in the cholinergic system in the striatum following vestibular loss [59], this will be an important avenue for further study.

It is conceivable that the sampling paradigm used in this experiment was not sensitive enough to measure rapid changes in the electrical stimulation-evoked release of some neurotransmitters. Microdialysis required the collection of samples over min and therefore it is possible that rapid changes in neurochemical levels were masked by the average neurochemical levels over time. In the future, the use of a more time-specific sampling method would be beneficial. However, it should be noted that previous studies using 20 min sampling periods have successfully detected changes in neurotransmitter release in the brain following electrical stimulation of the vestibular system [44] and although this temporal resolution may not detect instantaneous changes, it should detect cumulative changes. For example, Horii et al. [44] detected significant changes in ACh release in the hippocampus following electrical stimulation of the round window.

In conclusion, this study presents the first analysis of amino acid and monoamine levels in the striatum, using microdialysis, following electrical stimulation of the rat peripheral vestibular system, designed to evoke vestibular nystagmus. The results show that the main effects of such stimulation were a decrease in serine and threonine levels, independently of the side of analysis, and a decrease in the DOPAC/DA ratio that was specific to the ipsilateral side of stimulation. Furthermore, taurine showed a complex decrease on the contralateral side in stimulated animals, that varied according to the current intensity. These results are consistent with the view that the vestibular system exerts significant effects on the function of the striatum, albeit in a complex way [3].

Financial Disclosure: This research was supported by grants from the University of Otago Research Committee (9076 to PFS*) and the Maurice and Phyllis Paykel Trust (1954 to PFS*). LS was supported by a University of Otago PhD Scholarship and YZ by a Jean Cathie Estate Senior Research Fellowship administered by the Auckland Medical Research Foundation.
